# An in-depth multi-omics analysis in RLE-6TN rat alveolar epithelial cells allows for nanomaterial categorization

**DOI:** 10.1186/s12989-019-0321-5

**Published:** 2019-10-25

**Authors:** Isabel Karkossa, Anne Bannuscher, Bryan Hellack, Aileen Bahl, Sophia Buhs, Peter Nollau, Andreas Luch, Kristin Schubert, Martin von Bergen, Andrea Haase

**Affiliations:** 1 Department of Molecular Systems Biology, Helmholtz-Centre for Environmental Research (UFZ), Permoserstraße 15, 04318 Leipzig, Germany; 2 Department of Chemical and Product Safety, German Federal Institute for Risk Assessment (BfR), Max-Dohrn-Straße 8-10, 10589 Berlin, Germany; 3 Institute of Energy and Environmental Technology (IUTA) e.V, Bliersheimerstraße 58-60, 47229 Duisburg, Germany; 4 German Environment Agency, 06844 Dessau-Roßlau, Germany; 5 Research Institute Children’s Cancer Center and Department of Pediatric Hematology and Oncology, University Medical Center Hamburg-Eppendorf, 20251 Hamburg, Germany; 6 Institute of Biochemistry, Leipzig University, Brüderstraße 34, 04103 Leipzig, Germany

**Keywords:** Nanomaterials, Grouping, Proteomics, Metabolomics, SH2 profiling, WGCNA, Multi-omics

## Abstract

**Background:**

Nanomaterials (NMs) can be fine-tuned in their properties resulting in a high number of variants, each requiring a thorough safety assessment. Grouping and categorization approaches that would reduce the amount of testing are in principle existing for NMs but are still mostly conceptual. One drawback is the limited mechanistic understanding of NM toxicity. Thus, we conducted a multi-omics in vitro study in RLE-6TN rat alveolar epithelial cells involving 12 NMs covering different materials and including a systematic variation of particle size, surface charge and hydrophobicity for SiO_2_ NMs. Cellular responses were analyzed by global proteomics, targeted metabolomics and SH2 profiling. Results were integrated using Weighted Gene Correlation Network Analysis (WGCNA).

**Results:**

Cluster analyses involving all data sets separated Graphene Oxide, TiO2_NM105, SiO2_40 and Phthalocyanine Blue from the other NMs as their cellular responses showed a high degree of similarities, although apical in vivo results may differ. SiO2_7 behaved differently but still induced significant changes. In contrast, the remaining NMs were more similar to untreated controls. WGCNA revealed correlations of specific physico-chemical properties such as agglomerate size and redox potential to cellular responses. A key driver analysis could identify biomolecules being highly correlated to the observed effects, which might be representative biomarker candidates. Key drivers in our study were mainly related to oxidative stress responses and apoptosis.

**Conclusions:**

Our multi-omics approach involving proteomics, metabolomics and SH2 profiling proved useful to obtain insights into NMs Mode of Actions. Integrating results allowed for a more robust NM categorization. Moreover, key physico-chemical properties strongly correlating with NM toxicity were identified. Finally, we suggest several key drivers of toxicity that bear the potential to improve future testing and assessment approaches.

## Background

Engineered Nanomaterials (NMs) display many enhanced physico-chemical properties, rendering them interesting for various fields of applications, e.g. electronics, energy storage, medical devices, cosmetics or textiles [1]. But also conventional materials, such as pigments, silicates and other fillers, are considered as NMs by the European definition of NMs for regulatory purposes [2–4], that is now also integrated in the revised REACH legislation [5]. NMs can be taken up by organisms via different exposure routes, from which the lung is considered to be the most critical entry portal [6]. A significant fraction of inhaled NMs can reach the alveoli, which contain different cell types. Alveolar macrophages are responsible for clearing foreign material by phagocytosis. Many studies have investigated cellular uptake of NMs into various macrophage models and subsequent responses [7–10]. Alveolar epithelial cells, which represent the primary barrier between the gas phase and the bloodstream, are the most abundant cells. NM uptake into alveolar epithelial cells also has been demonstrated and may even lead to subsequent translocation of NMs into the systemic circulation in a size-dependent manner [6, 11]. Furthermore, it is known that lung epithelial cells serve as effectors to trigger immune and inflammatory processes in response to toxic stimuli. They can release chemokines and cytokines leading to the recruitment and activation of inflammatory cells. Thus, lung epithelial cells contribute mechanistically to lung tissue damage and inflammatory lung diseases like chronic pulmonary inflammation, asthma, emphysema and COPD [12–14].

NMs can be produced in a variety of variants by altering physico-chemical properties such as size or by applying chemical surface coatings [15, 16]. Each physico-chemical property may influence the interactions with biological systems e.g. toxicokinetics, cellular uptake and toxicity. Consequently, each single NM variant has to be assessed in a very complex, time- and cost-intensive procedure. It is virtually impossible to test the theoretically unlimited number of NM variants with respect to all relevant toxicological endpoints. Therefore, the development of NM grouping approaches for a more efficient assessment is indispensable [16–18]. For conventional chemicals grouping approaches are well established. A chemical category comprises a group of chemicals whose physico-chemical and (eco-) toxicological and/or environmental fate properties are likely to be similar or follow a regular pattern as a result of structural similarity [19]. Grouping of NMs is much more challenging as for instance demonstrating structural similarity requires more parameters. Moreover, several NM physico-chemical properties change during the life cycle due to agglomeration, dissolution, aging or interactions with biomolecules. Several approaches for grouping and categorizing of NMs have already been published using different strategies [20, 21]. Arts et al., for example, developed a tiered approach based on several intrinsic and system-dependent NM properties, biopersistence, biodistribution as well as cellular and apical toxic effects focusing on NM inhalation resulting in the assignment of NMs into four groups [22]. In this approach, after allocating soluble and biopersistent high aspect ratio NMs to group 1 and 2, respectively, the remaining NMs were assigned as “passive” (group 3) or “active” (group 4), depending on whether they show significant cellular effects and/or apical toxicity.

Grouping can serve multiple purposes determining the amount and type of information needed. For instance for NM prioritization less information is required compared to grouping for data gap filling for regulatory purposes. Knowledge on Mode of Actions (MoA) is not required but strongly facilitates grouping. Mechanistic information increases the confidence and renders the established groups more reliable. Thus, scientifically sound NM grouping approaches should consider NM MoA [18, 22, 23]. Mechanistic information also facilitates the establishment of adverse outcome pathways (AOP), which were introduced by the OECD to support regulatory decision-making [24]. Currently, Nano-AOP concepts are only beginning to emerge [25]. For instance, Halappanavar and co-workers suggested an AOP for pulmonary fibrosis [26].

Integrated multi-omics approaches and reliable statistical tools offer considerable opportunities to contribute to the development of AOPs and for establishing grouping criteria based on NM MoAs [27, 28]. Proteomics is the method of choice for the analysis of changes at the protein level and provides insights into cellular responses on both the regulatory and the executional level [29, 30]. On top of the abundance based analysis of proteins the analysis of posttranslational modifications, especially of phosphorylation at tyrosine residues, is closely linked to cell signaling and thus provides insights into the affected signaling pathways [31]. Metabolomics is the omics method closest to the phenotype of a biological system. Despite this, the use of metabolomics in nanotoxicology is relatively scarce [32]. While one omics method alone conveys a single section of the state of the cell or tissue, a combination of these techniques leads to a more global overview of cellular responses. Therefore, integration of results across multiple cellular response layers from various omics approaches results in higher confidence and allows for unravelling NM MoAs, establishing toxicity pathways and identifying key events. To establish mechanism based grouping it is necessary to obtain comprehensive omics data on a systematically selected panel of NM variants.

Here we conducted a multi-omics study involving 12 industrial relevant particles that fall under the European definition of NMs, covering different core materials like silica, titanium dioxide or phthalocyanines. Moreover, we systematically varied physico-chemical properties such as size, surface charge or surface hydrophobicity for silica-based NMs. The rat alveolar epithelial cell line RLE-6TN was chosen as a relevant cell model. Omics studies are providing a wealth of information on plenty of altered molecules individually but also on the integrated level about altered pathways. However, such alterations might be species-specific. Thus, we have chosen a rat cell model as we aimed to compare the outcome of this study with available in vivo data obtained in rats. Our integrated multi-omics approach comprised global proteomics, targeted metabolomics and tyrosine-specific phospho-proteomics by SH2 profiling. For the integrative analysis of proteomics and metabolomics data Weighted Gene Correlation Network Analysis (WGCNA) was used, which is a *p*-value independent co-expression network approach that can be used to explore the system-level functionality of genes or analytes [33]. Furthermore, WGCNA has shown to be a useful tool in systematically deciphering cellular responses or identifying critical pathways relevant to key traits or conditions [34, 35]. Additionally, this method allows the correlation with external conditions. Here we applied this approach for the first time to correlate molecular omics data to NM treatments and physico-chemical properties [35]. Moreover, this method enables the identification of trait specific key drivers that are functionally connected to particular traits, rendering them representative biomarker candidates.

The aim of this study was to identify NMs with similar MoAs based on overall integrated responses obtained from a multi-omics approach. Furthermore, correlations with physico-chemical properties as obtained by NM characterization in serum containing cell culture medium were investigated to select key properties contributing mainly to observed toxicity as well as to identify key drivers for NM toxicity, facilitating mechanistic based grouping as well as supporting future testing and assessment strategies.

## Results

### NM characterization

All NMs used in this study have been extensively characterized using various state-of-the arts methods. Table 1 summarizes key physico-chemical parameters. A comprehensive overview is given in the Additional file 7: Tables S1-S4 and includes a detailed characterization in serum containing F12K cell culture medium (Additional file 7: Tables S2-S4). Detailed characterizations of some of the NMs have also been published elsewhere [36–38].

**Table 1:** Overview on main physico-chemical properties

Name	Core Material	Surface Modification	PPS	Surface Area (BET)	Agglomerate Size in F12K (z.average)	Zeta Potential
			[nm]	[m^2^/g]	[nm]	[mV]
SiO2_15_Unmod	Silica	None	15	200	42	−35.5
SiO2_15_Phospho		Phosphonate	15	200	93	− 42.3
SiO2_15_Amino		Amino	15	200	144	−30.9
SiO2_40		None	40	50	255	− 38.8
SiO2_7		None	8	300	275	−26.6
SiO2_7_TMS2		2% TMS	8	300	159	−1.0
SiO2_7_TMS3		3% TMS	8	300	403	−8.8
TiO2_NM105	Titanium dioxide	None	21	51	3490	−16.5
Graphene Oxide	Graphene oxide	None	–	–	1927	−16.2
Phthalocyanine Blue	Cu-phthalo-cyanine Halogenated Cu-phathalo-cyanine	None	17	53	1760	−24.1
Phthalocyanine Green		None	–	69	1784	−37.0
Mn2O3	Manganese oxide	None	50	19.9	676	−24.6

Main physico-chemical properties are: core material, surface coatings/other modifications, primary particle size (PPS) as given by the manufacturer, NM surface area as determined by BET and provided by the manufacturer, agglomerate sizes in serum containing F12K as determined by DLS and Zeta Potential at pH 7.4

### Cell viability

Cell viability was assessed using WST-1 assay in RLE-6TN alveolar epithelial cells for all NMs after 24 h and 48 h for doses ranging from 7 to 112 μg/cm^2^, except for TiO2_NM105, which due to strong cytotoxicity was tested at 0.1–56 μg/cm^2^ only (Table 2). Only three of the tested NMs, TiO2_NM105, Mn2O3, and Graphene Oxide showed pronounced cytotoxic effects and reached an IC50 within the dose range tested. Most NMs induced either none or only weak cytotoxic effects. Particles were classified based on their cytotoxicity potency as high when an IC50 value was reached and low when an IC25 was achieved. If the cell viability did not decrease below 75% none was used as a classifier for overall cytotoxic potency.

**Table 2:** Overview on NM cytotoxicity

NM	IC50 [μg/cm^2^] 24 h	IC50 [μg/cm^2^] 48 h	IC25 [μg/cm^2^] 24 h	IC25 [μg/cm^2^] 48 h	Cytotoxic Potency
SiO2_15_Unmod	NR	NR	NR	NR	None
SiO2_15_Amino	NR	NR	NR	NR	None
SiO2_15_Phospho	NR	NR	NR	NR	None
SiO2_40	NR	NR	37	NR	Low
SiO2_7	NR	NR	38	56	Low
SiO2_7_TMS2	NR	NR	NR	NR	None
SiO2_7_TMS3	NR	NR	NR	NR	None
Phthalocyanine Blue	NR	NR	34	30	Low
Phthalocyanine Green	NR	NR	48	41	Low
TiO2_NM105	7	7	1	1	High
Mn2O3	28	NA	5	NA	High
Graphene Oxide	14	NA	7	NA	High

IC50 and IC25 values for 24 h and 48 h were given. Abbreviations NR: not reached; NA: not assessed

Kroll et al. have elaborated that in vivo overload conditions in rat lungs roughly correspond to in vitro doses of approximately 10 μg/cm^2^ [39, 40], which has been confirmed by others [41]. As nearly all of our NMs are tolerated up at 10 μg/cm^2^ we decided to use this dose for our multi-omics investigation. Only TiO2_NM105 shows a significant cytotoxicity at this dose, resulting in a cell viability of only 48%. This prompted us to additionally include two lower doses for TiO2 NM105, i.e. 0.1 μg/cm^2^ and 1 μg/cm^2^ in our study. However, as shown in Additional file 7: Figure S1 there were almost no significant changes observable for these lower doses neither in the proteome nor in the metabolome of RLE-6TNE cells. Therefore we decided to compare all NM treatments at 10 μg/cm^2^, despite the significant cytotoxicity for TiO2_NM105 at this dose. Nevertheless, when interpreting the results, the high cytotoxicity of TiO2_NM105 might be an issue and thus, should be considered accordingly.

### Proteomics, SH2 profiling and metabolomics

**Fig. 1:**
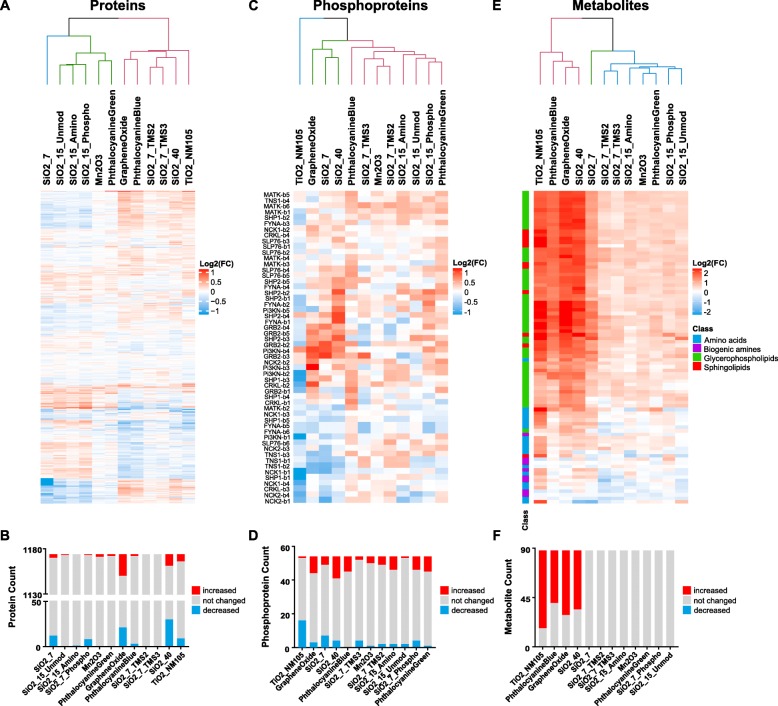
Summary of obtained proteins, phosphoproteins and metabolites. Presented are the results of the conducted Euclidean clustering analyses for proteins, phosphoproteins and metabolites in **a**, **c** and **e**, respectively, that are colored by Log2(FCs). **b**, **d** and **f** show the numbers of analytes that resulted in significantly (*p*-value ≤0.05) altered abundances for the respective data set

To get insights into molecular changes and NM MoAs, a multi-omics analysis was conducted in RLE-6TN cells. From global proteomics the fold-changes (FCs) of protein abundances relative to the control were obtained for 1174 proteins, which were quantified at least in triplicate over all the treatments. Initial hierarchical cluster analyses of protein FCs using the Euclidean distance measure revealed two main groups of NMs (Fig. 1a). The silica NMs SiO2_7, SiO2_15_Unmod, SiO2_15_Amino and SiO2_15_Phospho clustered together with Mn2O3 and Phthalocyanine Green, with the latter two having the least changes in protein abundances. On the other hand, there were Graphene Oxide, Phthalocyanine Blue, SiO2_7_TMS2, SiO2_7_TMS3, SiO2_40 and TiO2_NM105 which displayed an opposite protein expression pattern. Figure 1b illustrates that protein abundances were significantly increased or decreased (*p*-value ≤0.05) compared to control cells in case of treatment with SiO2_40, Graphene Oxide, SiO2_7 and TiO2_NM105. Thus, we assigned these four NMs as being “active” based on proteomics results. In contrast, for treatment with SiO2_7_TMS2, SiO2_7_TMS3, SiO2_15_Unmod, Mn2O3 and Phthalocyanine Green there were no significant changes observed.

SH2 profiling was performed to gain insights into changes of the state of tyrosine phosphorylation after NM treatment. Profiling was performed with 11 different SH2 domains that were selected based on previous unpublished studies out of 70 SH2 domains and considered to be most relevant to address NM mediated effects. The mean phospho-activity of a total number of 648 phosphoprotein bands was determined. The results of Far Western Blot analyses are shown in Additional file 7: Figure S2. Clustering of the SH2 profiles revealed separation of NMs into two major groups, where SiO2_40, Graphene Oxide, SiO2_7 and TiO2_NM105 were clearly separated from the other NMs (Fig. 1c). Significant (*p*-value ≤0.05) changes in the phosphorylation state of different proteins are summarized in Fig. 1d, demonstrating that SiO2_40 and Graphene Oxide are among the NMs leading to a strong increase in tyrosine phosphorylation while treatment with TiO2_NM105 resulted in a massive decrease in tyrosine phosphorylation of a large number of proteins. Consequently, SH2 profiling suggests SiO2_40, Graphene Oxide and TiO2_NM105 to be “active”. Increased tyrosine phosphorylation was primarily observed applying the SH2 domains of Pi3K, SLP76 and SHP2, respectively, indicating that the Pi3K-pathway, receptor signaling in general and MAPK signaling may be affected by NM treatment. In contrast, decreased tyrosine phosphorylation was preferentially detected by the SH2 domains of NCK1, NCK2 and TNS1 suggesting that NM treatment is associated with receptor signaling and cytoskeletal activity according to the main functional annotations of these SH2 domains.

To assess changes in the metabolome the AbsoluteIDQ p180 Kit (Biocrates) was applied allowing the quantification of 188 metabolites covering 5 different metabolite classes. 88 metabolites were identified at least in triplicate over all the treatments and hierarchical clustering of these compounds showed again a separation into two major groups. One group displayed few changes compared to control cells, recognizable by low FCs, whereas the other group induced many and strong FCs (Fig. 1e). Most alterations were caused by TiO2_NM105, Phthalocyanine Blue, Graphene Oxide and SiO2_40 (Fig. 1e and f). For these NMs observed changes were significant (*p*-value ≤0.05). SiO2_7 also caused alterations of several metabolites. However, here changes were not significant. Thus, we assigned TiO2_NM105, Phthalocyanine Blue, Graphene Oxide and SiO2_40 as “active” based on metabolomics results (Fig. 1e). SiO2_7 was considered as “equivocal” (Fig. 1e).

The metabolomic alterations were distributed over all metabolite classes such as lipids, amino acids (AAs) and biogenic amines, whereby the abundances across the different NMs were mainly consistent. Interestingly, lipids showed predominantly increased abundances compared to control cells, while AAs and biogenic amines were altered in both directions. However, the amount of these changes varied for the different NM treatments.

**Fig. 2:**
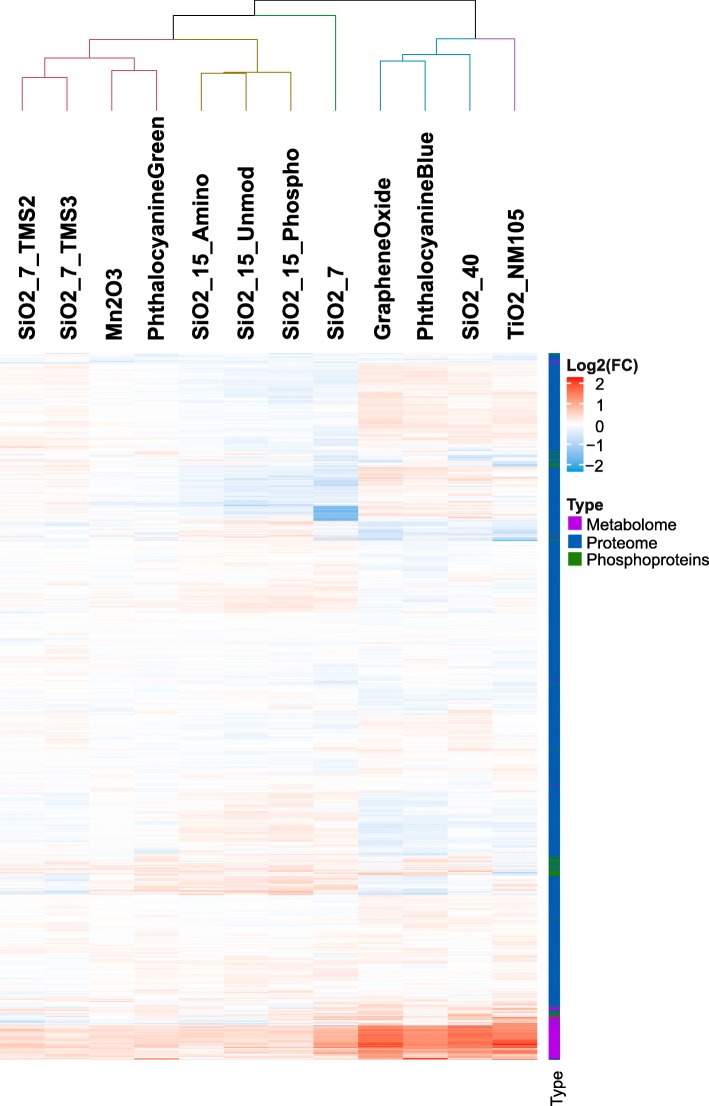
Results from integrative hierarchical clustering analysis. Presented is the result from a Euclidean clustering analysis conducted with protein, phosphoprotein and metabolite Log2(FCs) scaled to the same ranges (min = − 2, max = 2). Coloring was performed based on the scaled Log2(FCs)

An overall cluster analysis based on proteins, phosphoproteins and metabolites is presented in Fig. 2. For the overall analysis, all data sets were scaled to the same ranges to avoid a data set driven bias. Figure 2 shows that especially the four NMs that were mentioned above already, i.e. TiO2_NM105, Graphene Oxide, SiO2_40 and Phthalocyanine Blue, are grouped together, indicating that these are the “active” NMs based on overall analysis. SiO2_7 is outside this cluster but also clearly separated from the second large cluster as it showed significant alterations for proteins and phosphoproteins.

### Integrative analysis of proteomics and metabolomics results

**Fig. 3:**
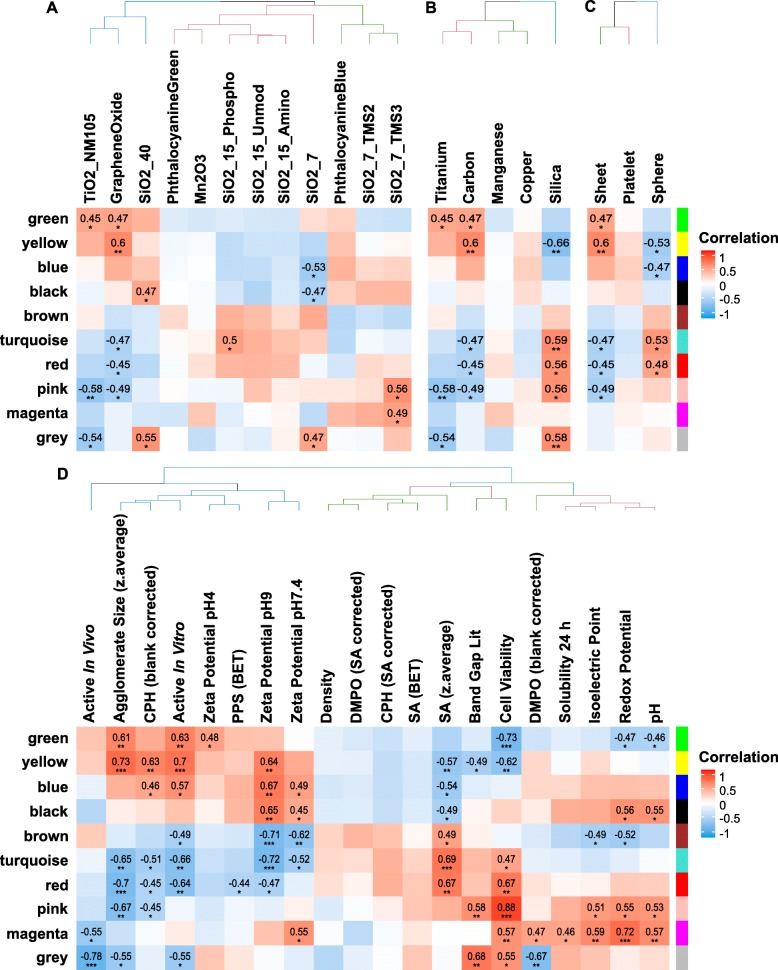
Results from module-trait correlation performed during WGCNA. Depicted are the modules of co-expressed analytes that were correlated to the NM treatments themselves (**a**), base materials (**b**), morphology (**c**) and physico-chemical properties (**d**), respectively. The heatmaps are colored by the correlation value and the significance of correlation is indicated by stars (*: *p*-value ≤0.1, **: *p*-value ≤0.05, ***: *p*-value ≤0.01)

For being able to combine the results of proteomics and metabolomics on the one hand and to relate the observed effects to other parameters such as physico-chemical properties on the other hand, a WGCNA was performed. Thus, co-expressed analytes (proteins and metabolites) were summarized into 10 modules, followed by a correlation of the obtained modules with traits such as the treatments themselves, core materials, morphology as well as physico-chemical properties and selected toxicological endpoints (Fig. 3). For each of the obtained modules significantly enriched pathways were determined using Ingenuity Pathway Analysis (IPA, Qiagen). A summary of results from WGCNA and IPA can be found in Additional file 7: Table S5, where the number of proteins and metabolites for each module as well as the results from the pathway enrichment analysis are listed.

The clustering of module-to-treatment correlations (Fig. 3a) revealed similar clustering of NMs as already identified from metabolomics, proteomics and SH2 profiling. On one hand, there were the “active” NMs TiO2_NM105, Graphene Oxide and SiO2_40 and on the other hand SiO2_15_Phospho, SiO2_15_Unmod and SiO2_15_Amino. Correlation with core materials (Fig. 3b) and morphology (Fig. 3c) also showed significant connections such as in case of silica NMs that significantly (*p*-value ≤0.05) correlated with the yellow, turquoise and grey modules. Importantly, the grey module contains all the analytes that could not be assigned to any of the other modules because of different expression patterns. Correlation patterns for the morphology show anti-correlation of sheets and spheres but for this comparison one has to keep in mind that Graphene Oxide was the only 1D NM in this study and thus this form is underrepresented. Consequently, the revealed differences were caused only by Graphene Oxide and might be attributed to other differences as well.

Interestingly, the linking of modules with physico-chemical properties and apical toxicological endpoints (Fig. 3d) allowed the identification of highly significant (*p*-value ≤0.05) correlations. Properties with most significantly correlating modules were agglomerate size and CPH reactivity as well as zeta potentials at pH 9, cell viability and redox potential. Furthermore, the classification into NMs that were shown to be “active” in vitro [37] or “active” in vivo [38] led to significant correlations with several modules. For more details the modules were analyzed further. Significant correlations with the grey module were neglected where appropriate.

**Fig. 4:**
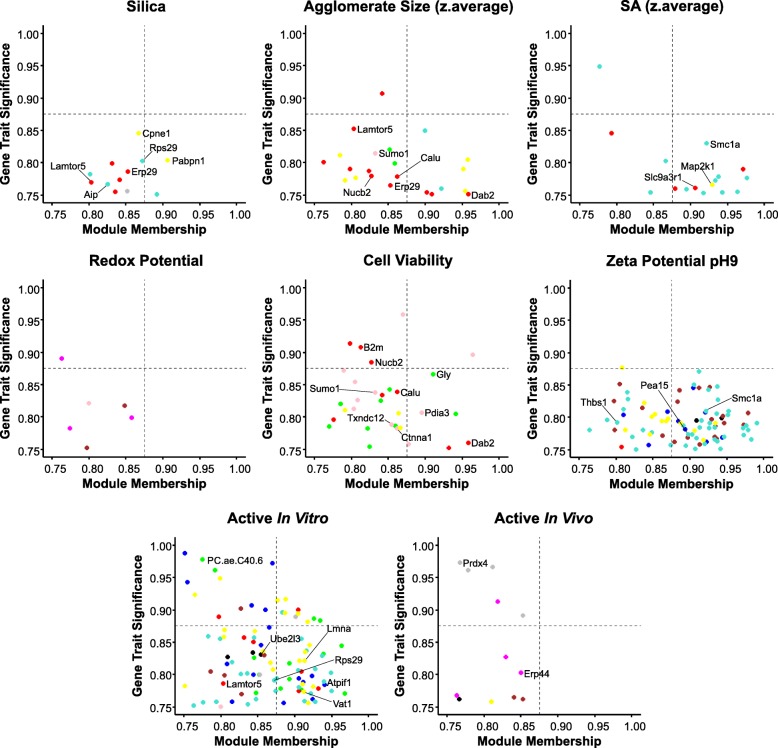
Identified key drivers for selected traits. Plotted are analytes that showed absolute gene significance ≥0.75 and absolute module membership ≥0.75 for the traits that showed highest correlation with at least one module. Analytes are colored based on the module they were assigned to during WGCNA. Names of analytes were added to the key drivers that were further described in the text

**Fig. 5:**
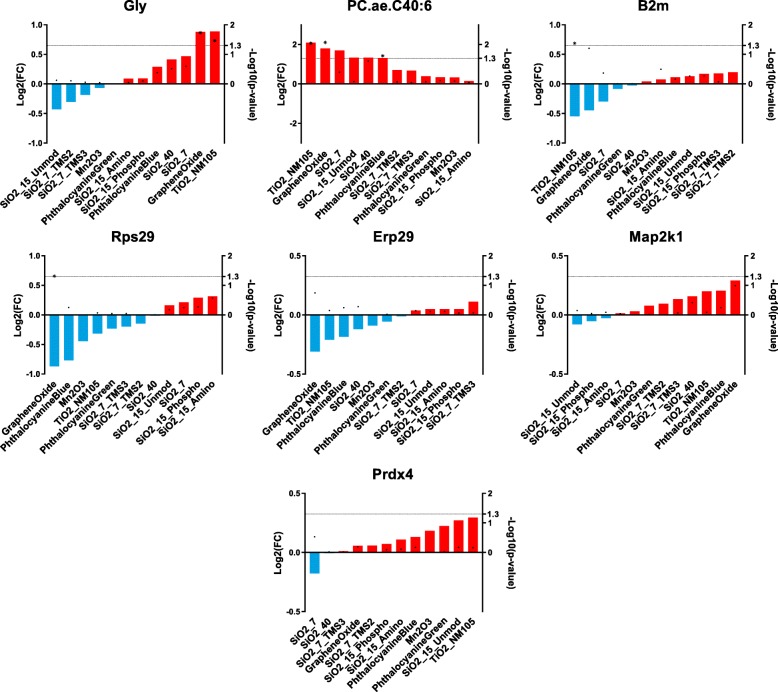
Summary of identified key drivers that allow distinguishing NM treatments. Shown are Log2(FCs) (left axis) for selected key drivers after NM treatment as well as obtained *p*-values (right axis) with respect to the control. Significantly (*p*-value ≤0.05) altered analytes are highlighted (*)

Furthermore, a key driver analysis was carried out for the mentioned traits (Fig. 4) that allowed the identification of analytes that were highly connected to the particular module and the significantly correlating trait because of their role as mediators of the observed effects. It was assumed that key drivers are those analytes with absolute gene significance ≥0.75 and absolute module membership ≥0.75. Gene Ontology (GO) terms of associated biological processes (BPs) were assigned to the selected key drivers to identify their functions. Figure 5 shows an overview of identified key drivers that allow differentiating between NM treatments. The trait-specific results from the key driver analysis are explained in the following section in detail.

### Silica-specific effects

Since different variants of silica NMs have been investigated in this study, we focused our data analysis in particular on silica-specific effects. Silica NMs showed highly positive correlations with the turquoise, red and pink modules (Fig. 3b). The analysis of these modules unraveled significant enrichment of pathways that are related to oxidative stress response (Additional file 7: Table S5) demonstrating that silica NMs tended to increase oxidative stress-related analytes. Negative correlation was observed for the yellow module that contains not only oxidative stress-related analytes but also apoptosis-related ones. Key drivers for silica-specific effects were analytes derived from the red, turquoise and yellow modules (Fig. 4). Annotation of GO BPs to these key drivers showed that Erp29 (endoplasmic reticulum protein 29, P52555), Lamtor5 (late endosomal/lysosomal adaptor, MAPK and MTOR activator 5, D3ZF11) and Rps29 (ribosomal protein S29, P62275) are regulators of apoptosis. In addition, Lamtor5, Aip (aryl-hydrocarbon receptor-interacting protein, Q5FWY5), Cpne1 (copine 1, D4A1R8) and Pabpn1 (poly(A) binding protein, nuclear 1, G3V7Z8) are known to respond to chemical stimuli. Consequently, all these biomolecules are biomarker candidates to assess NM MoAs.

### Agglomerate size and surface area dependent effects

Although the total number of NMs in our study is still low we nevertheless aimed to get first insights into the contribution of individual physico-chemical properties for observed biological outcome. Positive correlation of agglomerate size (Fig. 3d) was observed with the green and the yellow modules. Enriched pathways for these modules were mainly related to oxidative stress and apoptosis (Additional file 7: Table S5), suggesting that NMs forming large agglomerates resulted in higher abundances of analytes associated with these biological processes. Figure 3a demonstrates that this was especially true for TiO2_NM105 and Graphene Oxide, which showed significant correlation with at least one of the modules but also for SiO2_40 and Phthalocyanine Blue, which showed the same tendencies regarding their correlations with the green and the yellow modules. Phthalocyanine Green, which showed a similar agglomeration as Phthalocyanine Blue, behaved differently indicating that other parameters contribute as well. Negative correlations were observed for the turquoise, red and pink modules, which is true for the NMs mentioned above, whereas the silica NMs display a positive correlation with these. This suggests that mainly NMs with smaller agglomerate sizes as e.g. the silica NMs led to higher abundances for analytes assigned to these modules.

Key driver analysis (Fig. 4) revealed two proteins that already have been identified to be key drivers for silica-specific effects, namely Erp29 and Lamtor5, indicating that these proteins may be representative biomarkers for silica-specific toxicity assessment. Further key drivers that are related to stress response are Dab2 (DAB2, clathrin adaptor protein, O88797), Calu (Calumenin, G3V6S3), Nucb2 (nucleobindin 2, G3V8R1) and Sumo1 (small ubiquitin-like modifier 1, Q5I0H3), rendering them biomarker candidates as well.

Furthermore, the impact of the surface area was investigated, which showed anti-correlation to the agglomerate sizes. Additionally to the correlations that were already observed for the agglomerate sizes, the surface area showed correlation with the blue, black and brown module. The positive correlation with the brown module indicates that a large surface area is correlated to mitochondrial dysfunction (Additional file 7: Table S5). This was the case for the smaller agglomerates building NMs SiO2_15_Phospho, SiO2_15_Unmod, SiO2_15_Amino and SiO2_7. Related key drivers were identified from the red, yellow and the turquoise modules. Three of them are negatively regulating the cell cycle, namely Slc9a3r1 (SLC9A3 regulator, Q9JJ19), Map2k1 (mitogen activated protein kinase 1, Q01986) and Smc1a (structural maintenance of chromosomes 1A, Q9Z1M9).

### Zeta potential dependent effects

Positive correlations with the zeta potentials at pHs 7.4 and 9 were observed for the yellow, blue and black modules (Fig. 3d) that contain analytes related to oxidative stress response, apoptosis, detoxification and endocytosis (Additional file 7: Table S5). This indicates that NMs having a zeta potential closer to 0, which also indicates an increased agglomeration tendency, led to higher abundances in case of analytes connected to these biological processes, which is the case for Graphene Oxide and TiO2_NM105.

NMs with a highly negative zeta potential and having stable dispersions, in contrast, led to increased abundances for analytes that were assigned to the brown, turquoise or red modules. These modules contained analytes connected to mitochondrial dysfunction, oxidative stress response and detoxification but not apoptosis. Examples were SiO2_15_Phospho, SiO2_15_Unmod, SiO2_15_Amino and SiO2_7.

The key driver identification was performed based on the zeta potential at pH 9 since only the correlation with the values at pH 9 was significant (*p*-value ≤0.05). However, the correlation pattern was similar to the zeta potential at pH 7.4. The conducted analysis revealed one protein that has been already identified as key driver for surface area dependent effects, namely Smc1a. Furthermore Pea15 (phosphoprotein enriched in astrocytes, Q5U318) and Thbs1 (thrombospondin 1, Q71SA3) were identified to be key drivers, both positively regulating extrinsic apoptosis.

### Cell viability dependent effects

In addition to the physico-chemical properties of the NMs, the results of the cytotoxicity assays were incorporated into the integrative analysis. Negative correlation indicates decreased cell viability and was observable for the green and the yellow module, which contain analytes related to oxidative stress response, detoxification, amino acid metabolism and tRNA charging as well as apoptosis. Significant positive correlations were identified for the turquoise, red, pink and magenta modules that mainly contain analytes connected to oxidative stress response and detoxification (Additional file 7: Table S5).

The comparison to the treatment-specific correlation (Fig. 3a) suggests that NMs with a high cytotoxic potency such as TiO2_NM105 and Graphene Oxide result in positive correlation with the modules containing analytes related to oxidative stress response and apoptosis.

Key driver analysis led to the assumption that especially AAs that were assigned to the green module are a valuable measure for cell viability. Interestingly, high AA abundances indicate low cell viability since cell viability showed negative correlation with the green module. Furthermore, several key drivers have been identified that were already listed as indicators of agglomerate size-dependent effects, namely Dab2, Calu, Nucb2 and Sumo1. The latter one seems to be highly connected to the TiO2_NM105 treatment since it has been identified as key driver for this treatment as well. Additional key drivers were identified, from which one is positively regulating receptor-mediated endocytosis, two are regulating cell redox homeostasis and other two are positively regulating extrinsic apoptotic signaling pathways. These proteins are B2m (beta-2 microglobulin, P07151), Pdia3 (protein disulfide isomerase family A, member 3, A0A0H2UHM5), Txndc12 (thioredoxin domain containing, B0BN97) and Ctnna1 (catenin alpha 1, Q5U302). Importantly, none of these molecules have been shown to be key drivers for any of the other physicochemical properties.

### Analytes correlating with classification into “active” in vitro

Next, we investigated to which extent the classification into “active” based on published in vitro assays [37] correlates with the different modules and with which physico-chemical properties it clusters. The matrix that was used for this correlation is shown in Additional file 7: Tables S6 and S7. Wiemann and co-workers classified NMs as “active” and “passive” based on responses in NR8383 alveolar macrophage cells and classified SiO2_15_Unmod and Phthalocyanine Blue as “active” while SiO2_15_Phospho and SiO2_Amino were classified “passive”. It turned out that in vitro activity groups correlated with the agglomerate size. Key driver analysis revealed that especially phosphatidylcholines are highly connected to the classification into “active” in vitro.

The positive correlation of activity in vitro with the green module that contains glycerophospholipids suggests that increased glycerophospholipid abundances indicate NM activity in vitro. Furthermore, some key drivers have been identified that have also been found to be key drivers for silica-specific effects. Examples are Lamtor5 and Rps29, from which Lamtor5 is also a key driver for agglomerate size dependent effects. Further key drivers for the classification into “active” in vitro are for example Atpif1 (ATPase inhibitory factor 1, Q03344), Lmna (lamin A/C, G3V8L3), Ube2l3 (ubiquitin-conjugating enzyme E2L 3, B2RZA9) and Vat1 (vesicle amine transport 1, Q3MIE4). All of them are connected to mitochondrial processes. Furthermore, Lmna seems to be highly connected to the Graphene Oxide treatment, since it has been identified as key driver for Graphene Oxide as well.

### Analytes correlating with classification into “active” in vivo

In addition to the classification into “active” in vitro, a comparison to “active” in vivo was performed based on previously published data [38]. Landsiedel and co-workers performed short-term inhalation studies (STIS) with 14 materials (including 13 NMs) at a concentration of 0.5 to 50 mg/m3 in rats and assigned the materials to four different potency groups. Based on in vivo STIS results TiO2_NM105 and SiO2_15_Unmod can be classified “active” while SiO2_15_Phospho, SiO2_15_Amino and Phthalocyanine Blue were “passive”. For TiO2_NM105, SiO2_15_Phospho and SiO2_15_Amino this is in very good agreement to the classification based on our multi-omics analysis. For the two other NMs that were also included in our study we observed differences. Phthalocyanine Blue was classified “active” in the overall analysis but mainly due to the results of the metabolome. SiO2_15_Unmod was “passive” in our study but “active” in vivo, which might be due to the choice of the cell model in our study. The correlation with the classification into “active” in vivo during WGCNA was conducted based on the correlation matrix depicted in Additional file 7: Tables S6 and S7. Interestingly, the classification into “active” in vivo shows similar correlation patterns in WGCNA as the classification into “active” in vitro. However, only the correlation with the grey module is significant (*p*-value ≤0.05) and it has to be kept in mind that the grey module contains only compounds that could not be assigned to any of the other modules. This module contains for instance analytes that are related to ubiquitinylation and endocytosis (Additional file 7: Table S5). This might be interesting for a follow-up analysis.

Key driver analysis revealed two proteins that are regulating the cell redox homeostasis, namely Erp44 (endoplasmic reticulum protein 44, Q5VLR5) and Prdx4 (peroxiredoxin 4, Q9Z0V5). These molecules could be investigated further and might be interesting biomarkers as well.

## Discussion

In order to get insights into NM MoAs and to facilitate the establishment of NM grouping approaches based on mechanistic information, three different omics techniques were applied for 12 different NMs in RLE-6TN alveolar epithelial cells treated at a dose of 10 μg/cm^2^ for 24 h. In total five NMs, namely SiO2_7, SiO2_40, TiO2_NM105, Graphene Oxide and Phthalocyanine Blue induced significant changes in at least one of the omics approaches and might thus be categorized as “active”. All other NMs were rather similar to untreated controls and might thus be considered “passive”. It should be emphasized that overall data integration of all available omics data (Fig. 2) leads to a higher confidence in categorization compared to considering only one omics approach alone. Our overall analysis involved 1174 proteins, 88 metabolites and 54 phosphoproteins. Nevertheless, the overall categorization is still mainly driven by phosphoproteins (Fig. 1c) and metabolites (Fig. 1e), which are closer to the phenotype of a cell. Nevertheless, the proteome results are important to unravel NM MoAs as for each NM a high number of proteins has been assessed, which facilitates the identification of affected pathways.

In most cases the results were very consistent across all omics techniques but a few exceptions remain such that a few NMs may require additional follow-up analyses. SiO2_7, for instance, induced several significant changes, in particular in the proteome and in SH2 profiling. However, it did not really cluster together with the other “active” NMs nor with the “passive” NMs. The grouping of NMs based on our omics in vitro results can be compared to the grouping based on available in vivo and in vitro results [17, 38, 40]. The following NMs have been investigated in vivo in STIS already: TiO2_NM105, SiO2_15_Unmod, SiO2_15_Amino, SiO2_15_Phospho, Phthalocyanine Blue and Graphene Oxide. Overall, our results are in good agreement with previously published in vitro [37] and in vivo data [38, 40]. TiO2_NM105 has been classified “active” in vitro and in vivo [37, 38], which correlates very well with the strong alterations observed in all three omics analyses. SiO2_15_Amino and SiO2_15_Phospho have been classified “passive” in vitro and in vivo [36, 38], which again correlates well to data in our study. For SiO2_15_Unmod we observe a discrepancy as this NM did not induce many alterations in our study but was classified “active” in vitro and in vivo before [37, 38]. However, the classification in vitro has been achieved using a rat alveolar macrophage cell line, NR8383, which is a phagocytic cell line and the majority of inhaled NMs reaching the alveolar region are taken up in alveolar macrophages [42]. This emphasizes the importance of using different cell models for in vitro studies. For Phthalocyanine Blue we also observe a discrepancy as this NM was classified “active” in vitro [37] but was “passive” in vivo [17]. In our study, it was also classified “active” but mainly due to responses in the metabolome. Furthermore, the observed alterations are significant but rather weak. It also has to be considered that Phthalocyanine Blue was investigated here as technical grade without further purification after synthesis. Finally, Graphene Oxide was classified “active” in our study but was found “passive” in in vivo STIS [40]. However, in this study the authors state that Graphene Oxide is mainly found in alveolar macrophages which then mediate a spontaneous clearance and again would support the need to study the responses of Graphene Oxide in a second cell model such as a macrophage cell line. Thus, a few NMs seem to require special attention and follow-up experiments using another cell model. Such calibration is important and eventually decides if the omics approaches enhance the predictivity of the testing strategy and grouping framework.

Another important pillar of this study was the assessment of relationships of omics data to physico-chemical properties by WGCNA. Using this approach, two groups of NMs were identified that are consistent with the two groups that were identified from metabolomics, proteomics and SH2 profiling (Fig. 2). Both groups showed changes in biological functions associated with endocytosis, detoxification and oxidative stress response, but importantly, only the first group containing the supposedly “active” NMs led to alterations in pathways like apoptosis, tRNA charging and synthesis of different AAs. Indeed, several publications confirm that NMs often induce oxidative stress and apoptosis in comparable cell models [43, 44]. Alterations of those pathways might, thus, be indicators of NM toxicity.

Furthermore, we could identify a few physico-chemical properties that showed significant correlations with in vitro omics alterations. In our study several of the supposedly “active” NMs tend to form larger agglomerates in serum-containing cell culture medium. Examples for this observation are TiO2_NM105, Graphene Oxide and Phthalocyanine Blue. Thus, in our study agglomerate size instead of primary particle size is a better correlating parameter with respect to observed toxicity in vitro. However, the set of NMs investigated in our study is rather small and we also observed a few exceptions such as SiO2_40 and SiO2_7, which do not form large agglomerates and also lead to several significant alteration. Thus, other factors such as chemical composition, the synthesis route, particle shape or surface modification also have to be considered. Most likely, one cannot assume to identify an individual physico-chemical parameter that alone can be made responsible for specific cellular responses. Nevertheless, we suggest that the analysis method used here, WGCNA, is in particular useful to unravel such correlations in particular when comparing heterogeneous data sets. WGCNA allows analyzing omics data sets alongside with treatments, material composition, outcome of conventional toxicity testing as well as physico-chemical parameters. All of these can be integrated into one analysis. This certainly facilitates unraveling new correlations that then can be confirmed in follow-up investigations.

The subsequent key driver analysis unveiled several interesting findings. Firstly, AAs appear to be reliable markers of cell viability, indicated by significantly increased abundances for the presumably “active” group of NMs. SiO2_40 and TiO2_NM105 in particular provide significantly increased abundances for Met, Phe, Pro and Tyr. Previous in vitro metabolomics studies also showed an increase in such AAs upon treatment with silica or CuO NMs [45, 46]. A possible explanation could be cellular degradation of proteins due to autophagy and then release of AAs in the surrounding medium. Thus, such AAs have been already proposed as early biomarkers for apoptosis [46].

Glycerophospholipids were also identified as possible markers for activity in vitro. With the exception of SiO2_7, all supposedly “active” NMs led to significantly increased abundances for lipids. Similar results have been previously described for CeO_2_, CuO and SiO_2_ [44]. Furthermore, it has been already shown that this refers partly to their function as signaling molecules, gene regulators or indirect parts in glucose homeostasis [47–49]. It is generally assumed that sphingomyelins are crucial for cell survival and proliferation [48]. Our data confirm that NMs seem to have a large influence on cellular lipid balance and that the cell’s lipidome is a major target. Nevertheless, our study also confirms that it is highly beneficial to investigate alterations on proteome level to get insights into the NM MoAs. From key driver analysis several proteins were found to be highly connected to particular traits (Fig. 5), rendering them representative biomarker candidates. One of these was Rps29 that regulates apoptosis. Importantly, increased Rps29 levels lead to decreased apoptosis signaling [50]. Another biomarker candidate is Erp29 that is a marker for endoplasmic stress. Also Map2k1 should be considered a suitable biomarker. For all these key driver candidates Graphene Oxide, Phthalocyanine Blue, TiO2_NM105 and SiO2_40 tended to be in one group regarding their abundances compared to the control, while SiO2_7, SiO2_15_Unmod, SiO2_15_Amino and SiO2_15_Phospho tended to be in another group. Importantly, most of the key drivers were not significant based on information obtained from one omics approach, suggesting that comprehensive data sets as used here are essential to identify them. Identification of key drivers might facilitate future, more targeted assessment of NM toxicity.

Regarding the results of the systematically selected silica variants it appears that all SiO2_15 variants induce similar biomolecular changes in alveolar epithelial cells. In contrast, the hydrophobic silica variants SiO2_7_TMS2 and SiO2_7_TMS3 and the hydrophilic SiO2_7 and SiO2_40 were allocated to different clusters. Taken together the results for the different silica variants can be attributed to a) different routes of synthesis and b) surface modifications. All the SiO2_15 variants are precipitated silica while the others are pyrogenic (i.e. “fumed” silica). In general, pyrogenic silicas are considered more reactive, in particular in vitro [51]. In addition to the synthesis route also the chemical surface coating is very important. The surface-coated hydrophobic silica variants (SiO2_7_TMS2 and SiO2_7_TMS3) did not cause significant alterations in any of our omics analyses, suggesting that surface modification can modify the cellular responses of silica. This is in line with observed differences in vitro for SiO2_15_Unmod, SiO2_15_Amino and SiO2_15_Phospho in NR8383 macrophages [37], which also showed strongest responses for the unmodified silica while the surface modified variants were less toxic.

## Conclusion

In this study, a broad set of systematically selected NMs was investigated applying a multi-omics approach. Most importantly, all conducted omics analyses, namely proteomics, metabolomics and SH2 profiling revealed similar clustering of NMs, showing that results from the applied omics approaches were consistent. Furthermore, integrative analysis of all available omics data resulted not only in a more comprehensive data set but also leads to a higher confidence in categorization compared to considering only one omics approach alone.

Our data analysis strategy, based on WGCNA, allowed not only to get information about affected pathways and thus for insights into NM MoAs but also regarding correlation of physico-chemical properties with NM toxicity and/or alterations in omics data sets. The most important physico-chemical properties based on our data set were agglomerate size (but not primary particle size) and zeta potential. However, other parameters seem to be important as well. For instance the synthesis route seems to play a role at least for in vitro responses of silica. Pyrogenic silica NMs showed more alterations compared to precipitated silica NMs. However, our study also confirms the role of surface modification that can significantly modulate the activity of silica NMs.

Overall, our classification based on integrated in vitro alterations in different omics layers correlated well with published in vitro and in vivo results. However, a few NMs could not be correctly predicted (Graphene Oxide, SiO2_15_Unmod, Phthalocyanine Blue). This might be overcome in future by considering responses in other cell models. In particular one should consider responses in an alveolar macrophage model. Moreover, it would be useful to include human cell models in a follow-up analysis.

In summary, we have conducted a very systematic multi-omics in vitro study involving a well-selected set of NMs covering different core materials and involving specific changes in selected physico-chemical properties. We were able to obtain consistent results across all omics approaches. We also could identify potential biomarker candidates that might facilitate future hazard and risk assessment.

## Methods

### Nanomaterials (NMs)

For the present study a set of different NMs was selected from different classes but also variants of one core material with altered physico-chemical properties such as size and coating (Table 1). Three of them (SiO2_15_Unmod, SiO2_15_Amino, SiO2_15_Phospho) were provided by BASF SE, another two (Phthalocyanine Blue, Phthalocyanine Green) by BASF Colors and Effects, in technical grade. Four (SiO2_7, SiO2_40, SiO2_7_TMS2_ SiO2_7_TMS3) were manufactured by Evonik Industries, one (TiO2_NM105) was obtained from the JRC repository and one from Sigma-Aldrich (Graphene Oxide).

All NMs were delivered as powders with exception of the SiO_2_ NMs from BASF that were in suspension. All NM were fully characterized using stae-of-the art methodology as described elsewhere [36–38]. In addition all NMs were shown to be endotoxin-free using Limulus Amebocyte Lysate Endochrome test.

### NM dispersion

To disperse the NMs for in vitro studies, an indirect probe sonication protocol was used with a Bandelin Cup Horn (Bandelin, Germany). A 0.5 mg/ml stock solution was prepared in water or cell culture medium without serum. The centrifuge vial (2–50 ml) was placed in the middle of the Cup Horn or in a multi vial holder. Then, the Cup Horn was filled with water (continuous exchange) and the suspension was sonicated to a final power of 6 W (100%, 23 min). FCS was added afterwards to a final concentration of 10%.

Hydrophobic NM dispersions additionally contained 100 μg/ml of Pluronic F108 (Sigma-Aldrich, # 542342, Germany). Stock solutions were prepared and diluted in cell culture medium directly before application.

### Cell culture

RLE-6TN alveolar epithelial cells (ATCC, CRL-2300, USA) were cultured in F12 medium (Thermo Fischer Scientific, # 11765054, USA) supplemented with 2 mM L-glutamine (Pan Biotech, P04–80100, Germany), 0.01 mg/ml bovine pituitary extract (Thermo Fischer Scientific #13028014, USA), 0.005 mg/ml insulin (Sigma-Aldrich, # I0516, Germany), 2.5 ng/ml insulin-like growth factor (Sigma-Aldrich, # I3769, Germany), 0.00125 mg/ml transferrin (Sigma-Aldrich, # T1147, Germany), and 2.5 ng/ml epidermal growth factor (Sigma-Aldrich, # E4127, Germany), 10% fetal bovine serum (heat-inactivated, PAN Biotech, P30–1506, Germany), 100 U/mL penicillin and 100 μg/mL streptomycin (PAN Biotech, P06–07100, Germany). Cells were detached using 2.5 ml Accutase solution (Sigma Aldrich, A6964-500ML, Germany) and sub cultivated with a ratio of 1:5 twice a week.

### Study design and dosimetry

The applied concentration of 10 μg/cm^2^ was selected based on a similar rationale as described in Kroll et al., who concluded that in vivo overload conditions in rat lungs should correspond to in vitro doses of approximately 10 μg/cm^2^ [39]. Thus, we considered this dose as realistic for in vitro studies and in-line with corresponding in vivo studies, as also concluded by others [41]. The only exception was TiO2_NM105 due to its high cytotoxic potential. Therefore, effects upon TiO2_NM105 exposure were also investigated at doses of 0.1 μg/cm^2^ and 1 μg/cm^2^. The results for these lower doses are presented in Additional file 7: Figure S1, which shows that there were almost no significant changes observable neither in the proteome nor in the metabolome. Thus, also TiO2_NM105 was investigated at a dose of 10 μg/cm^2^ in the conducted screening.

In proteome first changes may already occur a few hours (3–5 h) after treatment. Typically, changes are expected to occur gradually covering a time frame of 3 h–48 h, or sometimes even later. In contrast, changes in the metabolome and in particular in the lipidome typically require longer and are usually first detected after 24 h. Thus, 24 h was regarded as a good compromise for covering both, changes in proteome and metabolome.

For each treatment 5 biological replicates were performed. Each biological repeat included untreated control samples.

### Cell viability assay

To determine cytotoxic effects caused by the different NMs the WST-1 assay (Roche, Switzerland) was performed in accordance to the manufacturer’s instructions. Briefly, 1 × 10^4^ cells per well were seeded into 100 μl/well of a 96-well plate. After 24 h cells were treated with freshly dispersed NMs in respective concentrations. Following the incubation period of 24 h or 48 h, the supernatants were removed and washed twice with PBS. The WST-1 reagent was mixed with fresh medium (1:10), put onto the cells and incubated for 1 h. Since some NMs cause interferences at 450 nm, the manufacturer’s procedure was slightly modified. After incubation with the dye, all supernatants were transferred into a new 96-well plate to leave behind NMs. Finally, the plate was analyzed at 450 nm. Triton-X100 (1%) served as positive control and non-treated cells as negative control.

### Sample preparation

For in vitro sample preparation each biomolecule was extracted from a distinct cell culture dish. 4 × 10^6^ RLE-6TN cells were seeded into a 60 cm^2^ cell culture dish (TPP, Switzerland) for metabolite and protein extraction. The next day, cells were treated with 10 μg/cm^2^ freshly dispersed NMs in complete cell culture medium for 24 h. To extract metabolites, cells were firstly washed twice with PBS, detached with Accutase and counted to normalize metabolite concentrations afterwards. Then, 1 ml of the extraction medium (EM) containing 5% chloroform, 45% methanol, 50% water was added. The mixtures were rotated (30 min, 4 °C), centrifuged (10 min, 500 x g, 4 °C) and the supernatants were dried under vacuum followed by storage at − 20 °C. Finally, metabolites were resuspended directly before metabolomics kit preparation.

Proteins were collected using RIPA buffer containing 0.05 M Tris/ HCl (pH 7.4, Roth, Germany), 0.15 M NaCl (Roth, Germany), 0.001 M EDTA (Roth, Germany), 1% Igepal (Sigma Aldrich, Germany), 0.25% Na-deoxycholate (Sigma Aldrich, Germany), 10 mM Na-Pyrophosphate (Sigma Aldrich, Germany), 10 mM β-Glycerolphosphate (Sigma Aldrich, Germany), 1 mM Sodiumorthovanadate (Sigma Aldrich, Germany). Before use the following components were added: 10 μl/ml Protease-inhibitor (Merck Millipore, USA), 10 μl/ml β-Mercaptoethanol, 10 μl/ml NaF and 2 μl/ml Na-Pervanadate (received from the reaction of 16 μl H_2_O_2_ with 100 μl Sodiumorthovanadate for 30 min at RT). Cells were washed three times with PBS before addition of 1 ml extraction buffer. Dishes were shaken (10 min, 4 °C), cell debris were collected with a cell scraper. To improve cell lysis, samples were frozen at − 80 °C, thawed and rotated for 30 min at 4 °C. After centrifugation (30 min, 12,000 x g, 4 °C), the protein concentration was determined using Bradford assay (Bio-Rad, USA).

### Targeted metabolomics

To determine the quantity of the extracted metabolites the AbsoluteIDQ p180 Kit (Biocrates, Austria) was used and conducted as described earlier [52]. In brief, metabolite pellets were resolved in 85% EtOH (99.8%, Sigma Aldrich, Germany) / 15% PBS (0.1 M, Sigma Aldrich, Germany). The volume of the extraction solvent was adjusted to the counted cell number: 2–4 × 10^6^ cells – 75 μl, 5–7 × 10^6^ cells – 150 μl, 8–10 × 10^6^ cells – 225 μl. During re-solvation, the tubes were shaken (1200 rpm, 20 min, RT) and vortexed responsively for three cycles. The kit preparation was conducted according to manufactures instructions. Briefly, 10 μl of each extract as well as internal and calibration standards were added to the filter of the 96-well kit plate and dried using nitrogen. Then, metabolites were derivatized with PITC (Sigma Aldrich, Germany) and extracted using 5 mM ammonium acetate (Sigma Aldrich, Germany) in MeOH (AppliChem, Germany). Extracts for HPLC-MS/MS and FIA-MS/MS were diluted separately. The final extracts were analyzed by an API 5500 triple quadrupole mass spectrometer (ABSciex, Germany) coupled with an Agilent 1260 Infinity HPLC system (Agilent, USA). Resulting spectra were analyzed using Analyst® software and MetIDQ provided by the kit. At the end, metabolite concentrations were normalized to the respective cell numbers. Studies have shown that the cell number of different cell lines correlates well with the set of metabolites tested in the Biocrates p180 kit [53]. Values below LOD were not taken into account.

### Untargeted proteomics

For protein quantification a tandem mass tag (TMT)-labeling strategy (TMT-10-plex, Thermo Scientific, USA) was used. 50 μg protein of each sample were processed as specified in manufacturer’s instructions. Samples from biological replicates were combined and desalted using cartridges (SPEC PT C18AR, Agilent, USA). The LC-MS/MS analyses war performed as described earlier (eigen Referenz). In brief, samples were analyzed on a nano-UPLC system (Ultimate 3000, Dionex, USA) coupled online via a chip-based ESI source (Nanomate, Advion, USA) to a mass spectrometer (QExactive, Thermo Scientific, USA). After trapping (Acclaim PepMap 100 C18, 3 μm, nanoViper, 75 μm × 5 cm, Thermo Fisher, Germany), peptides were separated on a reversed-phase column (Acclaim PepMap 100 C18, 3 μm, nanoViper, 75 μm × 25 cm, Thermo Fisher, Germany), applying a non-linear gradient of 150 min. MS raw data were processed using ProteomeDiscoverer 2.1.0.81. The database search was performed against the UniprotKB/Swissprot protein database of *Rattus norvegicus* (28 April 2017, only reviewed entries). Peptide and protein false discovery rates (FDR) were set to 1%. Proteins with at least two identified peptides were kept and proteins were quantified based on the intensities of the top three identified peptides. This workflow resulted in fold changes (FCs, treatment vs. control) for 2290 proteins and the data were log2-transformed and median normalized afterwards.

### SH2 profiling

SH2 profiling was performed as described previously [31]. In brief, whole cellular extracts were separated by SDS-PAGE, transferred to PVDF membranes, blocked with 10% skim milk in TBST buffer and probed with different SH2 domains pre-complexed with streptavidin/horseradish-peroxidase conjugate at a concentration of 1 μg/ml. Tyrosine phosphorylated proteins were detected by chemiluminescence, films were scanned and signal intensities of individual phosphoprotein bands were quantified applying the ImageJ software package. Mean signal intensities of phosphoprotein bands were calculated from three to five biological replicates and fold changes of phosphorylation were determined in comparison to mean signals obtained from untreated or solvent treated cells.

### Statistical analysis

Statistical analysis of the log2-transformed FCs was performed in R-3.5.0. To unravel significant (*p*-value ≤0.05) changes compared to control the Student’s t-test was performed for analytes that were quantified in at least three of five biological replicates over all the treatments. This resulted in 1174 proteins and 88 metabolites that were used for further analyses. The obtained *p*-values were Benjamini & Hochberg adjusted. Hierarchical clustering was conducted with Euclidean distance measure and complete clustering algorithm. FCs and *p*-values for all data sets can be found in Additional file 1.

### Integrative weighted gene correlation network analysis (WGCNA)

FCs of proteins and metabolites that were quantified at least in triplicate over all the treatments were further analyzed using WGCNA. For this purpose the data were scaled to integer values between 0 and 100 and the data are shown in Additional file 2. The networks were constructed across all the measured samples with R [35, 54]. The used trait matrices, containing the NM treatments as well as core materials, morphology and physico-chemical properties of the tested NMs can be found in (Additional file 7: Tables S6-S10) together with the description of the physico-chemical properties (Additional file 7: Table S1) and the average values for all determined physico-chemical properties in FK12 (Additional file 7: Table S2-S4). These were further used for correlation analyses to allow for a better comparability with the in vitro situation. There, the presence of serum leads to the formation of protein coronas, which affect e.g. the agglomeration behavior of NMs as well as their uptake [55, 56]. Thus, physico-chemical properties assessed in FK12 were considered more relevant for the conducted correlation analyses than properties assessed in water.

For WGCNA, the soft power threshold was set to 18 to arrive at the network adjacency. The Topology Overlap Matrix (TOM) was created using a cut height of 0.1 and a minimum module size of 25. The analysis identified 10 modules of co-expressed analytes, identified with different colors (Additional file 7: Figure S3). The grey module contains all analytes that were not assigned to any of the other modules. A summary of analytes that have been assigned to each of the modules can be found in Additional file 3. Finally, for each of the obtained modules significantly enriched pathways were determined using IPA (Qiagen, Germany). The IPA core expression analysis was performed without setting a *p*-value threshold. All cell types were enabled except immune cells and immune cell lines. Lists of all enriched pathways for each module can be found in Additional file 4. The most interesting enriched pathways are summarized in Additional file 7: Table S5. Identification of trait specific key drivers was performed based on the WGCNA results. Therefore, for each analyte the module- and trait-specific gene significances and module memberships were calculated. Gene significances are obtained from the correlation of the analytes expression profiles with the respective trait. Module memberships are generated by correlating the analytes expression profiles with the module eigengenes that are defined as the first principal component of the module [57]. A summary of gene significances and module memberships can be found in Additional file 5. Key drivers are assumed to be analytes with absolute gene significance ≥0.75 and absolute module membership ≥0.75. For chosen traits GO terms of BPs were assigned to key drivers using the DAVID Bioinformatics Resources 6.8 [58] Functional Annotation with GOTERM_BP_ALL. Thus, key drivers that might be representative biomarkers for NM toxicity were identified. Annotated GO terms for the chosen traits can be found in Additional file 6.

## Supplementary information

**Additional file 1. **FCs and *p*-values obtained from proteomics, metabolomics and SH2 profiling.

**Additional file 2.** Scaled data from proteomics and metabolomics that have been used for WGCNA.

**Additional file 3.** Lists of analytes and their assignment to the obtained modules.

**Additional file 4.** Summary of enriched pathways for each module.

**Additional file 5.** Calculated module memberships and gene significances together with identified key drivers.

**Additional file 6.** To key drivers assigned GO terms of biological processes using the DAVID Bioinformatics Resources 6.8.

**Additional file 7.** Detailed NM characterization, Far Western Blots, WGCNA and IPA results.

## Data Availability

The datasets used and/or analyzed during the current study are available from the corresponding author on reasonable request.

## Abbreviations

AA: Amino Acid
AOP: Adverse Outcome Pathways
BPs: Biological Processes
FCs: Fold Changes
GO: Gene Ontology
IPA: Ingenuity Pathway Analysis
MoA: Mode of Actions
NM: Nanomaterial
PPS: primary particle size
STIS: Short-term Inhalation Studies
WGCNA: Weighted Gene Correlation Network Analysis
