# Supplementary material for: An in-depth multi-omics analysis in RLE-6TN rat alveolar epithelial cells allows for nanomaterial categorization
Source: Part Fibre Toxicol. 2019 Oct 25;16:38. doi: 10.1186/s12989-019-0321-5 (PMC6814995; doi:10.1186/s12989-019-0321-5)
Supplement: Supplementary file 7 — Additional file 7. Detailed NM characterization, Far Western Blots, WGCNA and IPA results. [file 12989_2019_321_MOESM7_ESM.docx]

Supporting Information

An in-depth multi-omics analysis in RLE-6TN rat alveolar epithelial cells allows for nanomaterial categorization

Isabel Karkossa^a*^, Anne Bannuscher^b*^, Bryan Hellack^c^, Aileen Bahl^b^, Sophia Buhs^d^, Peter Nollau^d^, Andreas Luch^b^, Kristin Schubert^a,e^, Martin von Bergen^a,f*^ and Andrea Haase^b*^

^a^ Helmholtz-Centre for Environmental Research (UFZ), Department of Molecular Systems Biology, Permoserstraße 15, 04318 Leipzig, Germany

^b^ German Federal Institute for Risk Assessment (BfR), Department of Chemical and Product Safety, Max-Dohrn-Straße 8-10, 10589 Berlin, Germany

^c^ Institute of Energy and Environmental Technology (IUTA) e.V., Bliersheimerstraße 58-60, Duisburg, Germany

^d^ Research Institute Children’s Cancer Center and Department of Pediatric Hematology and Oncology, University Medical Center Hamburg-Eppendorf, Hamburg, Germany

^e^ Kennedy Institute of Rheumatology, University of Oxford, Roosevelt Drive, Oxford, United Kingdom

^f^ Institute of Biochemistry, Leipzig University, Brüderstraße 34, 04103 Leipzig, Germany

* Authors contributed equally

**Corresponding author:**

Andrea Haase

E-Mail: [Andrea.Haase@bfr.bund.de](mailto:Andrea.Haase@bfr.bund.de)

Telephone: +49-30-184123423

# Table S1: Description of Physicochemical Properties

| Variable | | Description | Parameter | | Unit | | | Concentration |
| --- | --- | --- | --- | --- | --- | --- | --- | --- |
| CPH – ratio sample/ blank | | CPH reactivity sample to blank ratio | CPH reactivity | | sample to blank ratio | | | 0.25 mg/mL |
| DMPO – ratio sample/ blank | | DMPO reactivity sample to blank ratio | DMPO reactivity | | sample to blank ratio | | | 0.125 mg/mL |
| CPH – SA corrected | | CPH reactivity blank corrected per m² SA | CPH reactivity per specific surface area | | blank corrected value in a.u. | | | 0.25 mg/mL |
| DMPO - SA corrected | | DMPO reactivity blank corrected per m² SA | DMPO reactivity per specific surface area | | blank corrected value in a.u. | | | 0.125 mg/mL |
| density | | Density | Density | | kg/m³ | | | 0.5 mg/mL |
| PPS_prov | | Primary particle size_provider | Primary particle size diameter | | nm | | | 0.5 mg/mL |
| SA_PPS_prov | | Specifc surface area calculated from PPS_provider | Specific surface area | | m²/g | | | 0.5 mg/mL |
| SA_BET_prov | | Specific surface area from BET_provider | Specific surface area | | m²/g | | | 0.5 mg/mL |
| SA_BET_IUTA | | Specific surface area from BET_IUTA | Specific surface area | | m²/g | | |  |
| PPS_from BET_prov | | Primary particle size calculated from BET_provider | Primary particle size diameter | | nm | | | 0.5 mg/mL |
| PPS_from BET_IUTA | | Primary particle size calculated from BET_IUTA | Primary particle size diameter | | nm | | | 0.5 mg/mL |
| PPS_SEM_mode | | Primary particle size calculated from SEM | Primary particle size diameter _ mode | | nm | | | 0.5 mg/mL |
| PPS_SEM_mean | | Primary particle size calculated from SEM | Primary particle size diameter _ mean | | nm | | | 0.5 mg/mL |
| SA_from_PPS_SEM | | Specific surface area from PPS_SEM | Specific surface area | | m²/g | | | 0.5 mg/mL |
| ZP4 | | zetapotential at pH4 | Zetapotential | | mV | | | 0.5 mg/mL |
| ZP7,4 | | zetapotential at pH7,4 | Zetapotential | | mV | | | 0.5 mg/mL |
| ZP9 | | zetapotential at pH9 | Zetapotential | | mV | | | 0.5 mg/mL |
| IEP | | pH value for point of no charge | Isoelectric Point | | pH | | |  |
| pH | | pH value at initial situation | pH value | | mV | | | 0.5 mg/mL |
| RP | | redoxpotential by Pt cathode | Redoxpotential | | mV | | | 0.5 mg/mL |
| z.average | | Hydrodynamic diameter by DLS | z.average / hydrodynamic diameter | | nm | | | 0.5 mg/mL |
| D50 | | Hydrodynamic diameter by DLS | D50 / hydrodynamic diameter | |  | | | 0.5 mg/mL |
| PDI | | Polidispersityindex | PDI | | a.u. (0-1) | | |  |
| SA_from_z.average | | specific surface area calculated from z.average | Specific surface area | | m²/g | | | 0.5 mg/mL |
| SA_from_d50 | | specific surface area calculated fromd50 | Specific surface area | | m²/g | | | 0.5 mg/mL |
| BGLit | | Band Gap Lit | Band Gap Lit | | eV | | | 0.5 mg/mL |
| DMPO per SA of BET_p | | DMPO reactivity per SA of BET provider | DMPO reactivity | | sample to blank ratio / SA_BET | | | 0.5 mg/mL |
| CPH per SA of BET_p | | CPH reactivity per SA of BET provider | CPH reactivity | | sample to blank ratio / SA_BET | | |  |
| DMPO per SA of BET_iuta | | CPH reactivity per SA of BET IUTA | CPH reactivity | | sample to blank ratio / SA_BET | | |  |
| CPH per SA of BET_iuta | | CPH reactivity per SA of BET IUTA | CPH reactivity | | sample to blank ratio / SA_BET | | |  |
| AGS_SEM | | Agglomerate_Size from SEM | Agglomerate_Size diameter | | nm | | |  |
| SA_AGS_SEM | | Specific surface area of the agglomerate_Size from SEM | Specific surface area of agglomerate_Size | | m²/g | | |  |
| DMPO per SA of DLS | | DMPO reactivity per SA of DLS | DMPO reactivity | | sample to blank ratio / SA_DLS | | |  |
| CPH per SA of DLS | | CPH reactivity per SA of DLS | CPH reactivity | | sample to blank ratio / SA_DLS | | |  |
| DMPO per AGS SEM | DMPO reactivity per AGS SEM | | | DMPO reactivity | | sample to blank ratio / AGS SEM |  |  |
| CPH per AGS SEM | CPH reactivity per AGS SEM | | | CPH reactivity | | sample to blank ratio / AGS SEM |  |  |
| DMPO per SA of SA_AGS SEM | DMPO reactivity per SA of AGS SEM | | | DMPO reactivity | | sample to blank ratio / SA_AGS SEM |  |  |
| CPH per SA of SA_AGS SEM | CPH reactivity per SA of AGS SEM | | | CPH reactivity | | sample to blank ratio / SA_AGS SEM |  |  |

# Table S2: Means for physicochemical properties in F12K (part 1)

|  | CPH - sample to blank ratio | DMPO - sample to blank ratio | density | PPS_prov | SA_PPS_prov | SA_BET_prov | SA_BET_IUTA | PPS_from BET_prov | PPS_from BET_IUTA | PPS_SEM_mode | PPS_SEM_mean | uncert_PPS_SEM | SA_from_PPS_SEM |
| --- | --- | --- | --- | --- | --- | --- | --- | --- | --- | --- | --- | --- | --- |
| Graphene Oxide | 8.14 | 0.74 | 1.80 | NA | NA | NA | 220.23 | NA | 15.14 | 27.66 | 57.30 | 53.50 | 58.17 |
| Mn2O3 | 1.06 | 1.40 | 4.50 | 50.00 | 26.67 | 19.90 | 58.12 | 67.00 | 22.94 | NA | NA | NA | NA |
| Phthalocyanine Blue | 1.08 | 1.09 | 1.62 | 17.00 | 217.86 | 53.00 | 49.02 | 69.88 | 75.55 | 21.70 | 26.20 | 10.40 | 141.36 |
| Phthalocyanine Green | 1.07 | 0.96 | 2.14 | NA | NA | 69.00 | 61.49 | NA | 45.59 | 38.86 | 47.20 | 17.60 | 59.40 |
| SiO2_15_Amino | 0.36 | 0.70 | 2.65 | 15.00 | 150.94 | 200.00 | NA | 11.32 | NA | 12.40 | 16.00 | 6.70 | 141.51 |
| SiO2_15_Phospho | 0.42 | 0.80 | 2.65 | 15.00 | 150.94 | 200.00 | NA | 11.32 | NA | 13.00 | 18.20 | 9.50 | 124.40 |
| SiO2_15_Unmod | 0.48 | 259.78 | 2.65 | 15.00 | 150.94 | 200.00 | NA | 11.32 | NA | 11.90 | 15.90 | 7.50 | 142.40 |
| SiO2_40 | 0.38 | 0.68 | 2.65 | 40.00 | 56.60 | 50.00 | 34.21 | 45.28 | 66.19 | 64.57 | 71.30 | 19.90 | 31.76 |
| SiO2_7 | 0.32 | 0.56 | 2.65 | 8.00 | 283.02 | 300.00 | 248.60 | 8.00 | 9.11 | 15.40 | 17.50 | 5.60 | 129.38 |
| SiO2_7_TMS2 | 0.52 | 0.97 | 2.65 | 8.00 | 283.02 | 300.00 | 213.18 | 8.00 | 10.62 | 14.54 | 16.40 | 5.00 | 138.06 |
| SiO2_7_TMS3 | 0.92 | 1.11 | 2.65 | 8.00 | 283.02 | 300.00 | 198.40 | 8.00 | 11.41 | 12.70 | 14.40 | 4.50 | 157.23 |
| TiO2_NM105 | 0.33 | 1.20 | 3.89 | 21.00 | 73.45 | 51.00 | 297.00 | 30.24 | 27.06 | 15.70 | 18.30 | 7.30 | 84.29 |

# Table S3: Means for physicochemical properties in F12K (part 2)

|  | ZP4 | ZP7,4 | ZP9 | IEP | pH | RP | z.average | D50 | PDI | SA_from_z.average | SA_from_d50 | BGLit |
| --- | --- | --- | --- | --- | --- | --- | --- | --- | --- | --- | --- | --- |
| Graphene Oxide | 19.44 | -16.23 | -13.66 | 5.31 | 7.76 | 151.73 | 1927.23 | 1818.10 | 0.38 | 1.83 | 1.90 | NA |
| Mn2O3 | -24.79 | -24.61 | -36.46 | NA | 8.11 | 217.77 | 675.59 | 627.61 | 0.24 | 1.99 | 2.13 | 1.29 |
| Phthalocyanine Blue | -8.81 | -24.05 | -26.98 | 2.74 | 8.16 | 197.47 | 1760.09 | 1466.46 | 0.39 | 2.10 | 2.53 | NA |
| Phthalocyanine Green | -16.45 | -36.97 | -36.90 | 2.28 | 8.12 | 194.77 | 1783.92 | 1783.66 | 0.46 | 1.61 | 1.61 | NA |
| SiO2_15_Amino | 2.76 | -30.94 | -40.47 | 4.18 | 8.00 | 183.47 | 144.22 | 40.24 | 0.14 | 20.65 | 56.31 | 8.90 |
| SiO2_15_Phospho | -18.45 | -42.28 | -45.12 | 2.47 | 8.02 | 166.73 | 92.83 | 43.08 | 0.12 | 24.61 | 52.56 | 8.90 |
| SiO2_15_Unmod | -5.30 | -35.53 | -43.60 | 3.49 | 7.98 | 189.73 | 42.23 | 40.32 | 0.19 | 54.17 | 56.25 | 8.90 |
| SiO2_40 | -0.65 | -38.77 | -26.96 | 3.97 | 8.14 | 188.83 | 255.00 | 265.79 | 0.17 | 8.88 | 8.52 | 8.90 |
| SiO2_7 | 1.56 | -26.63 | -32.04 | 4.13 | 7.79 | 171.20 | 275.11 | 237.73 | 0.19 | 8.23 | 9.53 | 8.90 |
| SiO2_7_TMS2 | -1.49 | -1.04 | -1.86 | 336.71 | 8.02 | 354.77 | 159.40 | 128.42 | 0.12 | 14.59 | 17.65 | 8.90 |
| SiO2_7_TMS3 | -11.64 | -8.78 | -11.56 | 596.27 | 8.22 | 371.40 | 403.03 | 126.57 | 0.12 | 6.27 | 17.90 | 8.90 |
| TiO2_NM105 | -10.42 | -16.46 | -11.68 | NA | 7.88 | 206.77 | 3489.63 | 3044.06 | 0.53 | 0.50 | 0.54 | 3.20 |

# Table S4: Means for physicochemical properties in F12K (part 3)

|  | DMPO per SA of BET_p | CPH per SA of BET_p | DMPO per SA of BET_iuta | CPH per SA of BET_iuta | DMPO per SA of DLS | CPH per SA of DLS | Solubility_24h | Solubility_48h | Solubility_48h + F108 |
| --- | --- | --- | --- | --- | --- | --- | --- | --- | --- |
| Graphene Oxide | NA | NA | 0.003 | 0.037 | 0.428 | 4.700 | 0.010 | NA | NA |
| Mn2O3 | 0.070 | 0.053 | 0.024 | 0.018 | 0.711 | 0.535 | 0.010 | NA | NA |
| Phthalocyanine Blue | 0.021 | 0.020 | 0.022 | 0.022 | 0.517 | 0.511 | 0.010 | NA | NA |
| Phthalocyanine Green | 0.014 | 0.015 | 0.015 | 0.017 | 0.603 | 0.658 | 0.010 | NA | NA |
| SiO2_15_Amino | 0.004 | 0.002 | NA | NA | 0.045 | 0.023 | 1.110 | 1.950 | NA |
| SiO2_15_Phospho | 0.004 | 0.002 | NA | NA | 0.033 | 0.017 | 1.109 | 1.410 | NA |
| SiO2_15_Unmod | 0.006 | 0.002 | NA | NA | 0.023 | 0.009 | 2.010 | 4.235 | NA |
| SiO2_40 | 0.014 | 0.008 | 0.020 | 0.011 | 0.076 | 0.042 | 1.620 | 4.287 | NA |
| SiO2_7 | 0.002 | 0.001 | 0.002 | 0.001 | 0.069 | 0.039 | 4.930 | 9.387 | NA |
| SiO2_7_TMS2 | 0.003 | 0.002 | 0.005 | 0.002 | 0.069 | 0.037 | 8.450 | 11.487 | 5.300 |
| SiO2_7_TMS3 | 0.004 | 0.003 | 0.006 | 0.005 | 0.195 | 0.163 | 5.450 | 14.000 | 3.900 |
| TiO2_NM105 | 0.024 | 0.007 | 0.021 | 0.006 | 2.725 | 0.742 | 0.010 | NA | NA |

# Figure S1: Dose-dependent effects of TiO2_NM105 on metabolome and proteome


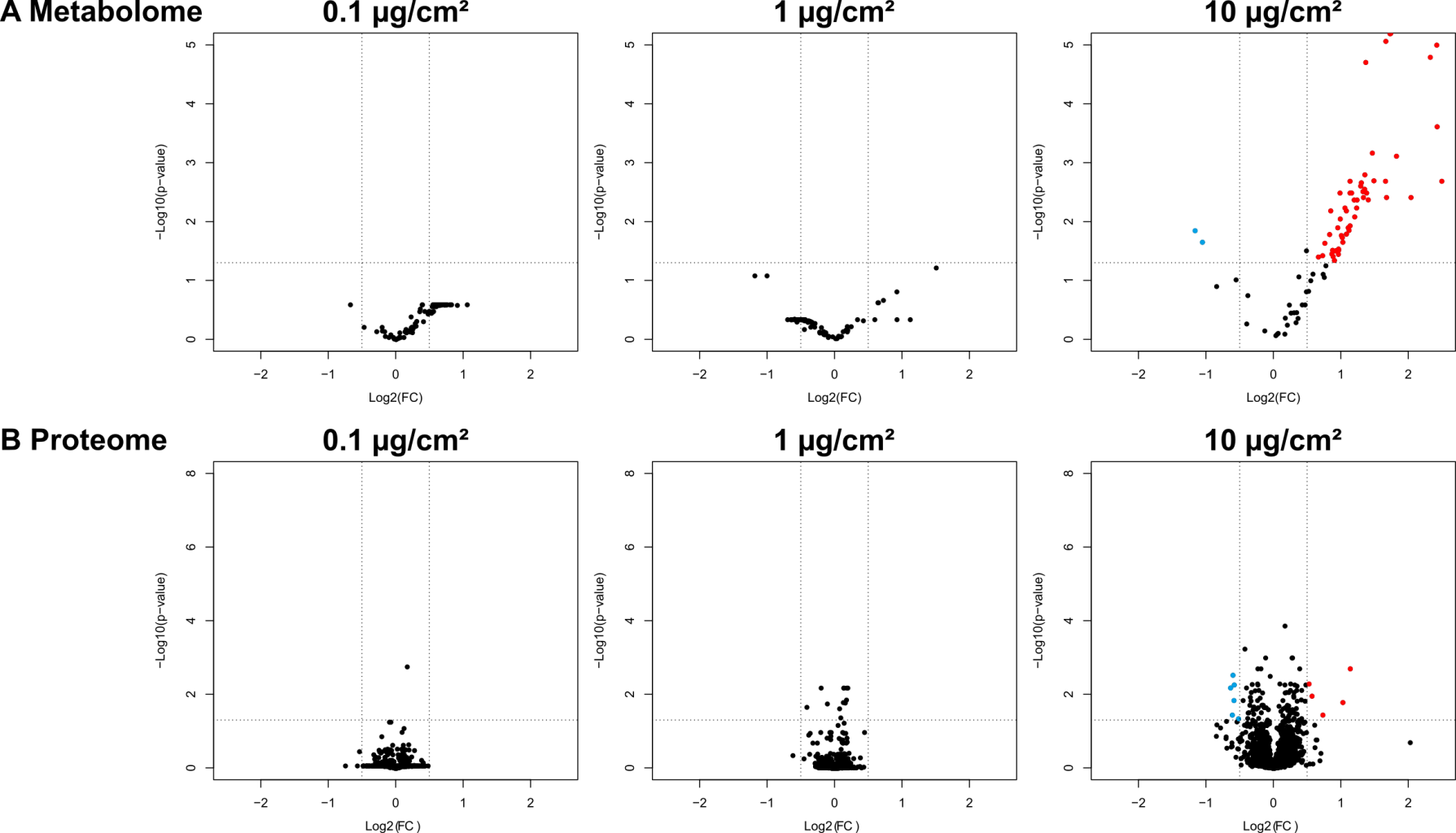


Depicted are results from the investigation of dose-dependent effects on the metabolome (A) and proteome (B) for TiO2 NM-105. Log2(FCs) and ‑Log10(adjusted p-values) for all analytes detected are presented.

# Figure S2: SH2 Profiling

Depicted are representative images obtained by Far Western Blot Analysis using different SH2 probes.

# Table S5: Results of WGCNA and Ingenuity Pathway Analysis (IPA)

Listed are the modules that were obtained from WGCNA as well the number of proteins (# Proteins) and the number of metabolites (# Metabolites) in each module. Furthermore list of enriched pathways from IPA is given for each module together with the corresponding -Log10(p-values) and the number of molecules (# Molecules) within the module that are related to the identified pathway. * indicates that z-scores are available for the pathway.

| **Module** | **# Proteins** | **# Metabolites** | **IPA Pathway** | **-Log10(p-value)** | **# Molecules** |
| --- | --- | --- | --- | --- | --- |
| **green** | 43 | 80 | tRNA Charging* | 2.02E+01 | 14 |
|  |  |  | Asparagine Biosynthesis | 5.74E+00 | 2 |
|  |  |  | Glutathione-mediated Detoxification | 2.15E+00 | 2 |
| **yellow** | 167 | 1 | Apoptosis Signaling* | 4.02E+00 | 6 |
|  |  |  | NRF2-mediated Oxidative Stress Response* | 3.80E+00 | 8 |
|  |  |  | ERK/MAPK Signaling* | 3.72E+00 | 8 |
|  |  |  | Caveolar-mediated Endocytosis Signaling | 3.56E+00 | 5 |
|  |  |  | tRNA Charging* | 3.27E+00 | 5 |
|  |  |  | Mitochondrial Dysfunction | 2.56E+00 | 6 |
|  |  |  | Sirtuin Signaling Pathway* | 2.53E+00 | 8 |
|  |  |  | Clathrin-mediated Endocytosis Signaling | 2.27E+00 | 6 |
|  |  |  | Oxidative Phosphorylation* | 1.97E+00 | 4 |
|  |  |  | Cell Cycle: G1/S Checkpoint Regulation | 1.80E+00 | 3 |
| **blue** | 203 | 0 | Glycolysis* | 3.85E+00 | 4 |
|  |  |  | Sirtuin Signaling Pathway* | 3.82E+00 | 11 |
|  |  |  | Epithelial Adherens Junction Signaling | 3.09E+00 | 7 |
|  |  |  | Caveolar-mediated Endocytosis Signaling | 3.04E+00 | 5 |
|  |  |  | PI3K/AKT Signaling* | 2.63E+00 | 6 |
|  |  |  | Superoxide Radicals Degradation | 2.50E+00 | 2 |
|  |  |  | Aryl Hydrocarbon Receptor Signaling | 2.46E+00 | 6 |
|  |  |  | Calcium Transport | 2.30E+00 | 2 |
|  |  |  | Mitochondrial Dysfunction | 2.16E+00 | 6 |
|  |  |  | tRNA Charging | 2.06E+00 | 3 |
|  |  |  | eNOS Signaling | 2.05E+00 | 6 |
|  |  |  | Lipid Antigen Presentation by CD1 | 1.84E+00 | 2 |
|  |  |  | Cell Cycle: G2/M DNA Damage Checkpoint Regulation | 1.77E+00 | 3 |
|  |  |  | Clathrin-mediated Endocytosis Signaling | 1.71E+00 | 6 |
| **black** | 89 | 1 | Remodeling of Epithelial Adherens Junctions | 8.03E+00 | 7 |
|  |  |  | Caveolar-mediated Endocytosis Signaling | 4.84E+00 | 5 |
|  |  |  | Glutathione Redox Reactions | 3.53E+00 | 3 |
|  |  |  | Epithelial Adherens Junction Signaling | 3.42E+00 | 5 |
|  |  |  | NRF2-mediated Oxidative Stress Response | 2.84E+00 | 5 |
|  |  |  | Clathrin-mediated Endocytosis Signaling | 2.77E+00 | 5 |
|  |  |  | Sirtuin Signaling Pathway* | 2.72E+00 | 6 |
| **brown** | 200 | 2 | Mitochondrial Dysfunction | 2.33E+00 | 6 |
| **turquoise** | 259 | 3 | Oxidative Phosphorylation* | 4.27E+00 | 8 |
|  |  |  | Mitochondrial Dysfunction | 3.50E+00 | 9 |
|  |  |  | Sirtuin Signaling Pathway* | 2.89E+00 | 10 |
|  |  |  | Tight Junction Signaling | 2.45E+00 | 7 |
|  |  |  | Cell Cycle: G2/M DNA Damage Checkpoint Regulation | 1.61E+00 | 3 |
|  |  |  | Aryl Hydrocarbon Receptor Signaling* | 1.51E+00 | 5 |
|  |  |  | ERK/MAPK Signaling | 1.45E+00 | 6 |
| **red** | 99 | 1 | GP6 Signaling Pathway* | 4.42E+00 | 6 |
|  |  |  | Remodeling of Epithelial Adherens Junctions | 1.44E+00 | 2 |
|  |  |  | Caveolar-mediated Endocytosis Signaling | 1.33E+00 | 2 |
| **pink** | 31 | 0 | Glutathione Redox Reactions | 4.80E+00 | 2 |
|  |  |  | Ketolysis | 4.03E+00 | 2 |
|  |  |  | Epithelial Adherens Junction Signaling | 1.66E+00 | 2 |
| **magenta** | 27 | 0 | Protein Ubiquitination Pathway | 2.26E+00 | 3 |
| **grey** | 56 | 0 | Clathrin-mediated Endocytosis Signaling | 3.43E+00 | 5 |
|  |  |  | Protein Ubiquitination Pathway | 2.11E+00 | 4 |
|  |  |  | Caveolar-mediated Endocytosis Signaling | 1.69E+00 | 2 |

# Table S6: Trait matrix (physicochemical properties, part 1)

|  | CPH - sample to blank ratio | DMPO - sample to blank ratio | Density | SA_BET_IUTA | PPS_BET_IUTA | ZP4 | ZP7.4 | ZP9 | IsoelectricPoint | pH |
| --- | --- | --- | --- | --- | --- | --- | --- | --- | --- | --- |
| GrapheneOxide | 8.14 | 0.74 | 1.80 | 220.23 | 15.14 | 19.44 | -16.23 | -13.66 | 5.31 | 7.76 |
| Mn2O3 | 1.06 | 1.40 | 4.50 | 58.12 | 22.94 | -24.79 | -24.61 | -36.46 | NA | 8.11 |
| PhthalocyanineBlue | 1.08 | 1.09 | 1.62 | 49.02 | 75.55 | -8.81 | -24.05 | -26.98 | 2.74 | 8.16 |
| PhthalocyanineGreen | 1.07 | 0.96 | 2.14 | 61.49 | 45.59 | -16.45 | -36.97 | -36.90 | 2.28 | 8.12 |
| SiO2_15_Amino | 0.36 | 0.70 | 2.65 | NA | NA | 2.76 | -30.94 | -40.47 | 4.18 | 8.00 |
| SiO2_15_Phospho | 0.42 | 0.80 | 2.65 | NA | NA | -18.45 | -42.28 | -45.12 | 2.47 | 8.02 |
| SiO2_15_Unmod | 0.48 | 1.17 | 2.65 | NA | NA | -5.30 | -35.53 | -43.60 | 3.49 | 7.98 |
| SiO2_40 | 0.38 | 0.68 | 2.65 | 34.21 | 66.19 | -0.65 | -38.77 | -26.96 | 3.97 | 8.14 |
| SiO2_7 | 0.32 | 0.56 | 2.65 | 248.60 | 9.11 | 1.56 | -26.63 | -32.04 | 4.13 | 7.79 |
| SiO2_7_TMS2 | 0.52 | 0.97 | 2.65 | 213.18 | 10.62 | -1.49 | -1.04 | -1.86 | 5.56 | 8.02 |
| SiO2_7_TMS3 | 0.92 | 1.11 | 2.65 | 198.40 | 11.41 | -11.64 | -8.78 | -11.56 | 12.80 | 8.22 |
| TiO2_NM105 | 0.33 | 1.20 | 3.89 | 57.00 | 27.06 | -10.42 | -16.46 | -11.68 | NA | 7.88 |

# Table S7: Trait matrix (physicochemical properties, part 2)

|  | Redox Potential | Agglomerate Size | SA Agglomerates | BandGapLit | DMPO - SA corrected | CPH - SA corrected | Solubility 24h | Cell Viability | Active *In Vitro* | Active *In Vivo* |
| --- | --- | --- | --- | --- | --- | --- | --- | --- | --- | --- |
| GrapheneOxide | 151.73 | 1927.23 | 1.83 | NA | 0.02 | 0.01 | 2.01 | 55.00 | NA | NA |
| Mn2O3 | 217.77 | 675.59 | 1.99 | 1.29 | 0.04 | 0.02 | 1.11 | NA | NA | NA |
| PhthalocyanineBlue | 197.47 | 1760.09 | 2.10 | NA | 0.03 | 0.02 | 1.11 | 90.00 | 1.00 | 0.00 |
| PhthalocyanineGreen | 194.77 | 1783.92 | 1.61 | NA | 2.73 | 0.74 | 0.01 | 88.00 | NA | NA |
| SiO2_15_Amino | 183.47 | 144.22 | 20.65 | 8.90 | 0.43 | 4.70 | 0.01 | 90.00 | 0.00 | 0.00 |
| SiO2_15_Phospho | 166.73 | 92.83 | 24.61 | 8.90 | 0.52 | 0.51 | 0.01 | NA | 0.00 | 0.00 |
| SiO2_15_Unmod | 189.73 | 42.23 | 54.17 | 8.90 | 0.60 | 0.66 | 0.01 | 98.00 | 1.00 | 1.00 |
| SiO2_40 | 188.83 | 255.00 | 8.88 | 8.90 | 0.08 | 0.04 | 1.62 | 95.00 | NA | NA |
| SiO2_7 | 171.20 | 275.11 | 8.23 | 8.90 | 0.07 | 0.04 | 4.93 | 82.00 | NA | NA |
| SiO2_7_TMS2 | 354.77 | 159.40 | 14.59 | 8.90 | 0.07 | 0.04 | 8.45 | 100.00 | NA | NA |
| SiO2_7_TMS3 | 371.40 | 403.03 | 6.27 | 8.90 | 0.19 | 0.16 | 5.45 | 100.00 | NA | NA |
| TiO2_NM105 | 206.77 | 3489.63 | 0.50 | 3.20 | 0.71 | 0.53 | 0.01 | 48.00 | 1.00 | 1.00 |

# Table S8: Trait matrix (treatments)

|  | SiO2_7_Hydrophil | SiO2_7_TMS2 | SiO2_7_TMS3 | SiO2_15_Unmod | SiO2_15_Amino | SiO2_15_Phospho | SiO2_40 | Mn2O3 | TiO2_NM105 | PhthalocyanineBlue | PhthalocyanineGreen | GrapheneOxide |
| --- | --- | --- | --- | --- | --- | --- | --- | --- | --- | --- | --- | --- |
| Graphene Oxide | 0 | 0 | 0 | 0 | 0 | 0 | 0 | 0 | 0 | 0 | 0 | 1 |
| Mn2O3 | 0 | 0 | 0 | 0 | 0 | 0 | 0 | 1 | 0 | 0 | 0 | 0 |
| Phthalocyanine Blue | 0 | 0 | 0 | 0 | 0 | 0 | 0 | 0 | 0 | 1 | 0 | 0 |
| Phthalocyanine Green | 0 | 0 | 0 | 0 | 0 | 0 | 0 | 0 | 0 | 0 | 1 | 0 |
| SiO2_15_Amino | 0 | 0 | 0 | 0 | 1 | 0 | 0 | 0 | 0 | 0 | 0 | 0 |
| SiO2_15_Phospho | 0 | 0 | 0 | 0 | 0 | 1 | 0 | 0 | 0 | 0 | 0 | 0 |
| SiO2_15_Unmod | 0 | 0 | 0 | 1 | 0 | 0 | 0 | 0 | 0 | 0 | 0 | 0 |
| SiO2_40 | 0 | 0 | 0 | 0 | 0 | 0 | 1 | 0 | 0 | 0 | 0 | 0 |
| SiO2_7 | 1 | 0 | 0 | 0 | 0 | 0 | 0 | 0 | 0 | 0 | 0 | 0 |
| SiO2_7_TMS2 | 0 | 1 | 0 | 0 | 0 | 0 | 0 | 0 | 0 | 0 | 0 | 0 |
| SiO2_7_TMS3 | 0 | 0 | 1 | 0 | 0 | 0 | 0 | 0 | 0 | 0 | 0 | 0 |
| TiO2_NM105 | 0 | 0 | 0 | 0 | 0 | 0 | 0 | 0 | 1 | 0 | 0 | 0 |

# Table S9: Trait matrix (core materials)

|  |  | Silica | Manganese | Copper | Titanium | Carbon |
| --- | --- | --- | --- | --- | --- | --- |
| GrapheneOxide |  | 0 | 0 | 0 | 0 | 1 |
| Mn2O3 |  | 0 | 1 | 0 | 0 | 0 |
| PhthalocyanineBlue |  | 0 | 0 | 1 | 0 | 0 |
| PhthalocyanineGreen |  | 0 | 0 | 1 | 0 | 0 |
| SiO2_15_Amino |  | 1 | 0 | 0 | 0 | 0 |
| SiO2_15_Phospho |  | 1 | 0 | 0 | 0 | 0 |
| SiO2_15_Unmod |  | 1 | 0 | 0 | 0 | 0 |
| SiO2_40 |  | 1 | 0 | 0 | 0 | 0 |
| SiO2_7_Hydrophil |  | 1 | 0 | 0 | 0 | 0 |
| SiO2_7_TMS2 |  | 1 | 0 | 0 | 0 | 0 |
| SiO2_7_TMS3 |  | 1 | 0 | 0 | 0 | 0 |
| TiO2_NM105 |  | 0 | 0 | 0 | 1 | 0 |

# Table S10: Trait matrix (morphology)

|  |  | Spherical | Platelet | Sheet |
| --- | --- | --- | --- | --- |
| GrapheneOxide |  | 0 | 0 | 1 |
| Mn2O3 |  | 1 | 0 | 0 |
| PhthalocyanineBlue |  | 0 | 1 | 0 |
| PhthalocyanineGreen |  | 0 | 1 | 0 |
| SiO2_15_Amino |  | 1 | 0 | 0 |
| SiO2_15_Phospho |  | 1 | 0 | 0 |
| SiO2_15_Unmod |  | 1 | 0 | 0 |
| SiO2_40 |  | 1 | 0 | 0 |
| SiO2_7_Hydrophil |  | 1 | 0 | 0 |
| SiO2_7_TMS2 |  | 1 | 0 | 0 |
| SiO2_7_TMS3 |  | 1 | 0 | 0 |
| TiO2_NM105 |  | 1 | 0 | 0 |

# Figure S3: Results from module detection
